# Elevated Release of Presynaptic Glutamate: The Potential Pathogenesis of Anti‐NMDAR Encephalitis‐Associated Seizures

**DOI:** 10.1111/cns.70585

**Published:** 2025-10-17

**Authors:** Hongmi Huang, Yifei Huang, Sijun Li, Ying Wu, Shengyu Yang, Yuan Wu

**Affiliations:** ^1^ Department of Neurology, The First Affiliated Hospital Guangxi Medical University Nanning Guangxi China

**Keywords:** action potentials, anti‐NMDAR encephalitis, glutamate, immunogenic peptide, miniature excitatory postsynaptic current, neuron, seizures

## Abstract

**Background:**

The pathogenesis of anti‐N‐methyl‐D‐aspartate receptor encephalitis (anti‐NMDAR encephalitis)‐associated seizures remains elusive.

**Methods:**

Mice were injected with GluN1_359–378_ peptide to construct a model of anti‐NMDAR encephalitis. Next, the expression of NMDAR antibodies (NMDAR‐Ab) was detected in serum samples. The electroencephalograms (EEGs) of mice were recorded. Afterward, neuronal action potentials (APs) and miniature excitatory postsynaptic currents (mEPSCs) were examined following exposure to serum derived from model mice. The expression levels of subunits of the NMDA receptor (GluN1, GluN2B) and glutamate vesicular transporter 1 (Vglut1) were quantified via Western blot analysis. Additionally, mice were injected with pentylenetetrazol (PTZ) to construct an in vivo model of status epilepticus (SE). Lastly, neurons exposed to mouse serum were incubated in a magnesium‐free solution (Mg^2+^‐free) for 1, 2, and 3 h, and APs were assessed.

**Results:**

Following the administration of immunogenic peptide GluN1_359–378_, serum NMDAR‐Ab was detected. EEG recording revealed that 68.75% (11/16) of mice receiving GluN1_359–378_ exhibited epileptiform discharges. Moreover, the frequency of neuronal APs and mEPSCs following exposure to serum derived from mice receiving GluN1_359–378_ was increased. The surface expression level of the GluN1 protein was significantly decreased, whereas that of the total Vglut1 protein was increased. Moreover, the seizure latency of mice receiving GluN1_359–378_ was significantly shortened. Finally, after 1 to 2 h of incubation with Mg^2+^‐free solution, the frequency of neuronal APs following exposure to serum derived from mice receiving GluN1_359–378_ was increased.

**Conclusion:**

To the best of our knowledge, this is the first study to demonstrate that increased glutamate release may increase seizure susceptibility in patients with anti‐NMDAR encephalitis.

## Graphical abstract

Mice were injected with GluN1_359–378_ peptide to construct a model of anti‐NMDA receptor encephalitis. In anti‐NMDA receptor encephalitis, increased glutamate release may underlie the susceptibility of mice to seizures.

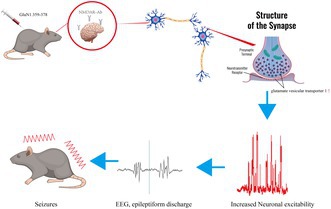

**Abbreviations**
anti‐NMDAR: anti‐N‐methyl‐D‐aspartate receptor
APs: action potentials
EEG: electroencephalograms
mEPSCs: miniature excitatory postsynaptic currents
Mg^2+^‐free: magnesium‐free solution
NMDAR‐Ab: NMDA receptor antibody
PTZ: pentylenetetrazol
SE: status epilepticus
Vglut1: glutamate vesicular transporter 1

## 1 Introduction

Anti‐N‐methyl‐D‐aspartate receptor encephalitis (anti‐NMDAR encephalitis) refers to an autoimmune disease of the central nervous system (CNS) that was first identified in female encephalitis patients with ovarian teratoma [1]. After immune activation induced by tumor‐associated antigens or other mechanisms, the synthesized antibodies cross the blood–brain barrier through compromised regions, with activated B cells infiltrating the brain and differentiating into plasma cells, thereby promoting the synthesis of N‐methyl‐D‐aspartate receptor (NMDAR) antibodies [2, 3]. Anti‐NMDAR encephalitis typically manifests as progressive neurological and psychiatric symptoms and positive levels of NMDAR antibodies (NMDAR‐Ab) in serum and cerebrospinal fluid [4, 5]. The majority of patients with anti‐NMDAR encephalitis develop seizures as the initial or prominent symptom, a condition referred to as anti‐NMDAR encephalitis‐associated seizures. However, the underlying pathogenesis remains to be elucidated [4, 6].

NMDARs are ionic glutamate receptors that are primarily distributed in neurons and mediate neuronal excitatory functions [7]. They are tetramer ion channels composed of various subunits, including GluN1, GluN2, and GluN3 [8]. GluN1 is the basic functional unit of NMDAR and plays a decisive role in cell membrane receptors [9]. GluN2 is an auxiliary unit that modulates physiological functions such as NMDAR agonist affinity, single‐channel conductance, and ion permeability [10]. GluN1, which cannot bind to GluN2, exists as a monomer, leading to receptor retention failure at the cell membrane surface and rapid degradation [11, 12]. Notably, NMDAR activation exclusively occurs following the binding of GluN1 with GluN2, which plays a critical role in regulating calcium ion inflow [13]. The binding of anti‐NMDAR antibodies with NMDARs on the neuronal membrane promotes the internalization of NMDARs, resulting in a reduction in the expression level of NMDARs on the cell membrane surface, thus contributing to symptoms associated with nervous system damage [14]. Previous studies have established that antibodies targeting GluN1 can alter the structure or function of postsynaptic NMDARs, resulting in the downregulation of NMDARs, eventually culminating in anti‐NMDAR encephalitis [15, 16].

Increased neuronal excitation and decreased neuronal inhibition are considered the classical mechanisms underlying seizures [17, 18]. Previous studies have demonstrated that NMDAR, which regulates neuronal excitatory functions, plays a key role in seizures [19]. Up‐regulating the expression of NMDARs can promote the development of seizures, whereas down‐regulation effectively alleviates seizures [20]. Nevertheless, the decrease in NMDAR activity is the primary pathogenesis underlying anti‐NMDAR encephalitis [14, 15, 16]. Therefore, the currently understood pathogenesis of anti‐NMDAR encephalitis does not account for the occurrence of seizures.

N‐methyl‐D‐aspartate receptors (NMDARs) play a pivotal role in regulating neuronal excitability and synaptic transmission, with their dysfunction closely implicated in seizure pathogenesis. Physiologically, presynaptic NMDARs act as feedback regulators to limit excessive glutamate vesicle exocytosis, maintaining the balance of excitatory neurotransmission via Ca^2^‐dependent signaling [8]. This regulatory role is critical for preventing neuronal hyperexcitability, a hallmark of seizures.

In anti‐NMDAR encephalitis, autoantibody‐induced NMDAR internalization reduces their surface receptor expression [1], disrupting the presynaptic feedback loop and disinhibiting glutamate release. Uncontrolled presynaptic glutamate then overactivates postsynaptic α‐amino‐3‐hydroxy‐5‐methyl‐4‐isoxazolepropionic acid receptors (AMPARs), the primary mediators of rapid excitatory transmission [21], thereby enhancing neuronal firing and lowering seizure thresholds.

This mechanism resolves the paradox in anti‐NMDAR encephalitis: while postsynaptic NMDAR hypofunction impairs synaptic plasticity, presynaptic glutamate disinhibition directly promotes hyperexcitability [19]. The observed increases in miniature excitatory postsynaptic currents (mEPSCs) and vesicular glutamate transporter 1 (Vglut1) expression levels support elevated presynaptic glutamate release as a key link between NMDAR dysfunction and seizure susceptibility.

To further elucidate the pathogenesis of anti‐NMDAR encephalitis‐associated seizures, a stable anti‐NMDAR encephalitis model should be constructed. Previous studies have noted depressive behavior, anhedonia, and memory deficits in animals injected with cerebrospinal fluid (CSF) or IgG antibodies derived from patients with anti‐NMDAR encephalitis [22, 23]. However, these models are regarded as passive immune models that can merely demonstrate the pathogenicity of anti‐NMDAR antibodies but fail to simulate autoantibody production [24]. Therefore, an active immune model that more closely reflects disease progression is warranted to explore the pathogenesis of anti‐NMDAR encephalitis, especially its association with seizures [25]. Active immune animal models can simulate antibody production in autoimmune diseases and play an instrumental role in investigating the pathogenesis of autoimmune encephalitis [26, 27]. Autoreactive antibodies targeting NMDAR recognize specific regions of its amino‐terminal domain (ATD) [3], encompassing the key amino acids asparagine and glycine at positions 368/369 of the protein [28], thereby promoting the immune effects of the disease. In the present study, mice were immunized with ATD peptides for 2 weeks to generate an anti‐NMDAR encephalitis model and investigate the potential mechanism underlying anti‐NMDAR encephalitis‐associated seizures. We hypothesize that elevated presynaptic glutamate release may underlie the susceptibility to seizures in anti‐NMDAR encephalitis, which could reconcile the paradox between reduced NMDAR activity and increased neuronal excitability observed in this disease.

## 2 Materials and Methods

### 2.1 Animals

A total of 36 C57BL/6 mice (8 weeks old, 20–25 g) and 12 newborn Sprague–Dawley (SD) rats (24 h old) were purchased from the Animal Experiment Center of Guangxi Medical University, China. Mice injected with peptides to construct the encephalitis models were assigned to a negative control (NC) group (*n* = 10), a control group (*n* = 10), and a GluN1_359–378_ group (*n* = 16), with an equal number of males and females in each group. Mice in the NC group were used for paraffin‐embedded tissue sectioning. Newborn rats were used for neuronal cell culture.

### 2.2 Design of Peptide

Previous research has established that the N368 and G369 amino acids of GluN1 amplify the immune effect of anti‐NMDAR encephalitis [28, 29]. Therefore, the immunogenic peptide GluN1_359–378_ (RKLVQVGIYNGTHVIPNDRK) was used in this study [29]. Specifically, GluN1_359–378_ was mixed with a Freund's Complete Adjuvant (CFA) containing *Mycobacterium tuberculosis* H37Ra (3 mg/mL) at a 1:1 volume ratio to prepare a GluN1_359–378_ emulsifier with a final peptide concentration of 1 mg/mL GluN1_359–378_ [29].

### 2.3 Construction of the Anti‐NMDA Receptor Encephalitis Model

Mice in the CluN1_359–378_ group were subcutaneously injected with a GluN1_359–378_ emulsifier (200 μL) to induce an autoimmune response. In contrast, mice in the control group were subcutaneously injected with 200 μL of a mixture of CFA and phosphate buffer saline (PBS) (1:1). Mice in the NC group were injected with 200 μL saline at the corresponding location. Then, behavioral observations were conducted daily. The serum and hippocampal tissues of mice in the NC group (*n* = 10), control group (*n* = 10), and CluN1_359–378_ group (*n* = 16) were harvested on the 14th day for molecular biological analyses.

### 2.4 Electroencephalogram (EEG) Recording

Subcutaneous needle electrodes were implanted in the left frontal (Fp1‐Avf), right frontal (Fp2‐Avf), left temporal (T3‐Avf), and right temporal (T4‐Avf) regions of C57BL/6 mice, with the electrode on the forelimb serving as the ground reference. Electroencephalographic (EEG) monitoring was performed using the Nuocheng EEG system (Nuocheng Electric Co. Ltd., Shanghai, China). EEG recordings were performed for 30 min following PTZ injection. The paper speed and sensitivity were adjusted to 4.5 cm/s and 200 μV/cm, respectively, and the results were independently assessed by two epileptologists in a blinded manner.

### 2.5 Enzyme‐Linked Immunosorbent Assay (ELISA)

An ELISA kit (Shanghai Jianglai Industry, JL20420) was used to detect NMDAR‐Ab in mouse serum, and the results were expressed as either negative or positive.

### 2.6 Immunocytochemistry (ICC) and Immunohistochemistry (IHC)

Neuronal cultures were performed as described in a previous study [18]. Briefly, neurons were assigned to the PBS group, control group, NC group, GluN1_359–378_ group, and anti‐GluN1 antibody (GluN1) group. After fixation with 4% paraformaldehyde, permeabilization with 0.3% Triton X‐100, and blocking with 5% BSA, paraffin‐embedded sections were incubated with PBS, mouse serum, and primary antibodies. Neurons in the PBS, control, NC, GluN1_359–378_, and GluN1 groups were incubated with PBS, control mouse serum, NC mouse serum (dilution ratio 1:10), GluN1_359–378_ mouse serum (dilution ratio 1:10), and anti‐GluN1 antibodies (dilution ratio, 1:50, Boster, BS90951) overnight at 4°C, respectively. The ZSGB‐BIO, PV‐9001, and PV‐PV‐6002 test kits were employed for neuronal staining.

After isoflurane inhalation anesthesia, mice in the NC group were euthanized, and the brain tissues were extracted and prepared into paraffin‐embedded sections. The sections were divided into PBS group, control group, NC group, GluN1_359–378_ group, and anti‐GluN1 antibody (GluN1) group. After dewaxing, antigen retrieval, and blocking, paraffin sections were incubated with PBS, mouse serum, and primary antibodies. Paraffin‐embedded sections in the PBS, control, NC, GluN1_359–378_, and GluN1 groups were incubated with PBS, control mouse serum, NC mouse serum (dilution ratio 1:10), GluN1_359–378_ mouse serum (dilution ratio, 1:10), and anti‐GluN1 antibodies (dilution ratio 1:50, Boster, BS90951) overnight at 4°C. The ZSGB‐BIO, PV‐9001, and PV‐PV‐6002 test kits were employed for neuronal staining. Images were captured using a Leica optical microscope. Image J (4.11.18012.20201123) software was used to analyze cumulative optical density and positive cell counting.

### 2.7 Transfection of HEK293 Cells

The cell culture solution was composed of Dulbecco's Modified Eagle Medium/Nutrient Mixture F‐12 (DMEM) (11320033, Gibco) supplemented with 10% FBS, 1% Glutamax (35050061, Gibco), and 1% Penicillin–streptomycin (15140‐122, Gibco). HEK293 cells (SUNNCELL, SNL‐014) were cultured at a density of 1 × 10^7^ cells. Transfection was performed when the cell confluence reached 80%–90% and cells were evenly distributed. Next, the transfected cells were divided into the Cytomegalovirus negative control group (CMV‐NC), CMV‐GluN1 group, and CMV‐GluN2B group. Using NanoTrans 20TM transfection reagent (B1001, BIOMEDICAL), plasmids encoding GFP‐tagged GluN1 and GluN2B subunits (Geicylin) were transfected into HEK293 cells using liposomes.

### 2.8 Immunofluorescence (IF) Staining

The serum of mice was used as the primary antibody to perform IF experiments on transfected HEK293T cells. The transfected HEK293T cells were divided into five groups, namely the CMV‐NC+GluN1_359–378_ group (cells in the CMV‐NC group incubated with serum from GluN1_359–378_ mice), CMV‐GluN1+control group (cells in the GluN1 group incubated with serum from control mice), CMV‐GluN1+GluN1_359–378_ group (cells in the CMV‐GluN1 group incubated with serum from GluN1_359–378_ mice), CMV‐GluN2B + control group (cells in the CMV‐GluN2B group incubated with serum from control mice), and CMV‐GluN2B + GluN1_359–378_ group (cells in the CMV‐GluN2B group incubated with serum from GluN1_359–378_ mice). After fixation with 4% paraformaldehyde, permeabilization with 0.3% Triton X‐100, and blocking with 5% bovine serum albumin (BSA), the cells were incubated overnight with the pre‐defined serum at 4°C (dilution ratio, 1:10). On the following day, they were stained using a multiplex fluorescent immunohistochemical staining kit (abs50012, Absin) [30]. Transfected cells were visualized by green fluorescence, positive serum was indicated by red fluorescence, and nuclei were counterstained with blue fluorescence.

To further detect NMDAR‐Ab in the serum of mice, an IF experiment was conducted using primary neurons, and the cells were divided into two groups, namely the control group and the GluN1_359–378_ group. Neurons were stained using multiplex fluorescent immunohistochemical staining kits (abs50012, Absin). After fixation with 4% paraformaldehyde, permeabilization with 0.3% Triton X‐100, and blocking with 5% bovine serum albumin (BSA), the neurons were incubated overnight with the following primary antibodies: anti‐neuron‐specific enolase (NSE) antibody (dilution ratio, 1:50, BOSTER, A02930) and mouse serum (dilution ratio, 1:10). NSE was visualized by green fluorescence, positive serum was indicated by red fluorescence, and nuclei were counterstained with blue fluorescence.

### 2.9 Western Blot Assay

Total surface proteins were extracted from samples, and Western blot analysis was performed based on the method outlined in previous studies [17, 18]. The membranes were incubated with the following primary antibodies: anti‐GluN1 antibody (dilution ratio, 1:1000; BOSTER, BS90951), anti‐ATP1A1 antibody (dilution ratio, 1:1000, Proteintech, 14418‐AP), anti‐Vglut1 (dilution ratio, 1:1000, Servicebio, D151135), and anti‐GAPDH antibody (dilution ratio, 1:8000, Sangon, D110016). Following this, the membranes were incubated with HRP‐conjugated Goat Anti‐Rabbit IgG secondary antibody (dilution ratio, 1:10000, Sangon, D110058). The ChemiScope6000 gel imaging system was employed to visualize and capture the results.

### 2.10 Construction of the In Vitro Model of Anti‐NMDAR Encephalitis

Neurons cultured for 8 days were divided into the control group and the GluN1_359–378_ group. They were subsequently incubated with mouse serum (3 h, 37°C). Specifically, neurons in the control and GluN1_359–378_ groups were incubated with the serum derived from mice in the control and GluN1_359–378_ groups, respectively. Neuronal action potentials (APs) and miniature excitatory postsynaptic currents (mEPSCs) were recorded in whole‐cell mode. To investigate the relationship between anti‐NMDAR encephalitis and seizures, the two groups of cells were exposed to magnesium‐free (Mg^2+^‐free) extracellular fluid, and neuronal APs were recorded at 1, 2, and 3 h after exposure. The method for detecting APs was based on previous studies [17, 18]. The method for detecting mEPSCs was adapted from the study undertaken by Markus T. Sainio et al. [31].

### 2.11 Construction of Pentylenetetrazol (PTZ) Induced Anti‐NMDAR Encephalitis Mouse Status Epilepticus (SE) Model

Mice in the control (*n* = 6, half male and half female) and GluN1_359–378_ (*n* = 6, half male and half female) groups were intraperitoneally injected with PTZ to induce SE. The method for PTZ administration and the criteria for evaluating SE have been outlined in a previous study [18]. The incubation period for each sample was calculated based on seizure characteristics, that is, the interval between the first PTZ injection and the first seizure episode.

### 2.12 Statistical Analyses

Statistical analyses were performed using SPSS 23.0 software, and data were expressed as mean ± standard error (mean ± SE). Parametric data were analyzed by one‐way analysis of variance (ANOVA) followed by Tukey's honestly significant difference (HSD) test for post hoc multiple comparisons to control the family‐wise error rate (FWER). Non‐parametric data were analyzed using the Kruskal–Wallis test followed by Dunn's post hoc test. Figures were generated using GraphPad Prism 6.0 (GraphPad Software, USA) or ImageJ. *p* < 0.05 was considered statistically significant.

## 3 Results

### 3.1 Identification of NMDAR‐Ab in Mouse Serum

**FIGURE 1:**
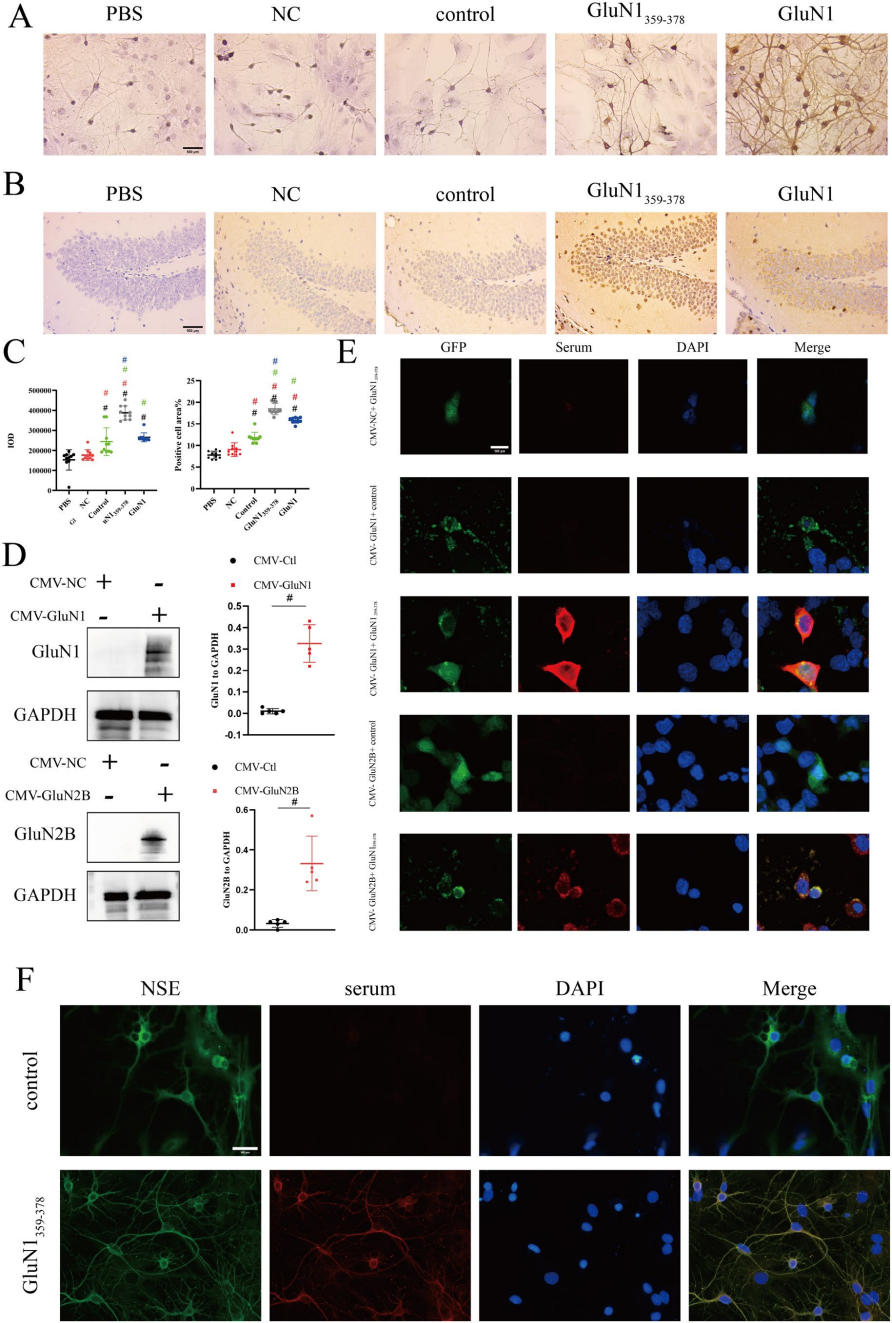
Identification of NMDAR‐Ab in the serum of mice with anti‐NMDAR encephalitis. (A) Results of ICC assay in neurons: Significant brown deposits were observed in neurons from the GluN1_359–378_ and anti‐GluN1 antibody groups (Scale: 500 μm). (B) IHC results of paraffin‐embedded sections: Brown deposition was noted in the hippocampal tissue from the GluN1_359–378_ and anti‐GluN1 antibody groups (Scale: 500 μm). (C) Results of IOD and positive cell area in the hippocampal area: A significant increase in IOD and positive cell area was detected in the GluN1_359–378_ group (*n* = 10, half male and half female; #, vs. PBS, *p* < 0.01; #, vs. NC, *p* < 0.01; #, vs. control, *p* < 0.01; #, vs. GluN1, *p* < 0.01). (D) Western blot results of transfected HEK293T cells: The expression level of GluN1 was significantly increased in the CMV‐GluN1 group. The expression of GluN2B was significantly increased in CMV‐GluN2 group (*n* = 5; #*p* < 0.01). (E) IF results of HEK293T cells post‐transfection with GluN1_359–378_; pronounced red fluorescence was detected in HEK293 cells incubated with serum derived from mice. In contrast, no red fluorescence was detected in HEK293 cells incubated with negative serum or control serum (scale: 100 μm). (F) IF results of neurons: Only green fluorescence was observed in neurons from the control group, whereas both green fluorescence and red fluorescence were observed in neurons from the GluN1_359–378_ group (Scale: 100 μm).

To demonstrate the pathogenicity of the immunogenic peptide GluN1_359–378_, NMDAR‐Ab was qualitatively detected in the serum of mice in each group using ELISA. The positive detection rate in the GluN1_359–378_, control, and NC groups was 68.75% (11/16), 10.00% (1/10), and 0 (0/10), respectively. As anticipated, the positive detection rate was significantly higher in the GluN1_359–378_ group compared to the control and blank groups (*p* < 0.05). In addition, significant tan deposits were observed in the GluN1 antibody and GluN1_359–378_ groups (Figure 1A). IHC results displayed brown‐yellow staining in the hippocampus of brain tissues incubated with serum derived from the GluN1_359–378_ and GluN1 groups, whereas no positive staining was detected in the PBS, control, and NC groups. These results validated that the GluN1_359–378_ peptide induced the production of autoantibodies that bind to antigens on neurons in mouse serum (Figure 1B). Afterward, the integral optical density (IOD) and positive cell area of IHC were calculated, revealing the most significant increase in positive cell area in the GluN1_359–378_ group (*p* < 0.01, Figure 1C). To further confirm the specificity of antibodies produced in mouse serum, HEK293T cells were transfected with plasmids encoding GluN1 and GluN2B subunits. Green fluorescence observed under the microscope indicated successful transfection. The transfection efficiency of HEK293T cells was subsequently evaluated by Western blot analysis, which revealed significant GluN1 expression in the CMV‐GluN1 group and significant GluN2B expression in the CMV‐GluN2 group, suggesting successful transfection (*p* < 0.01, Figure 1D). Subsequently, HEK293T cells transfected with GluN1 and GluN2B were incubated with the serum derived from mice. After incubation of HEK293T cells transfected with GluN1 and GluN2B with the serum collected from mice in the GluN1_359–378_ group, red fluorescence was observed (Figure 1E). Conversely, only green fluorescence was observed in the control group, whereas both green fluorescence and red fluorescence were observed in the GluN1_359–378_ group (Figure 1F).

### 3.2 Increased Neuronal Excitability in Mice With Anti‐NMDAR Encephalitis

**FIGURE 2:**
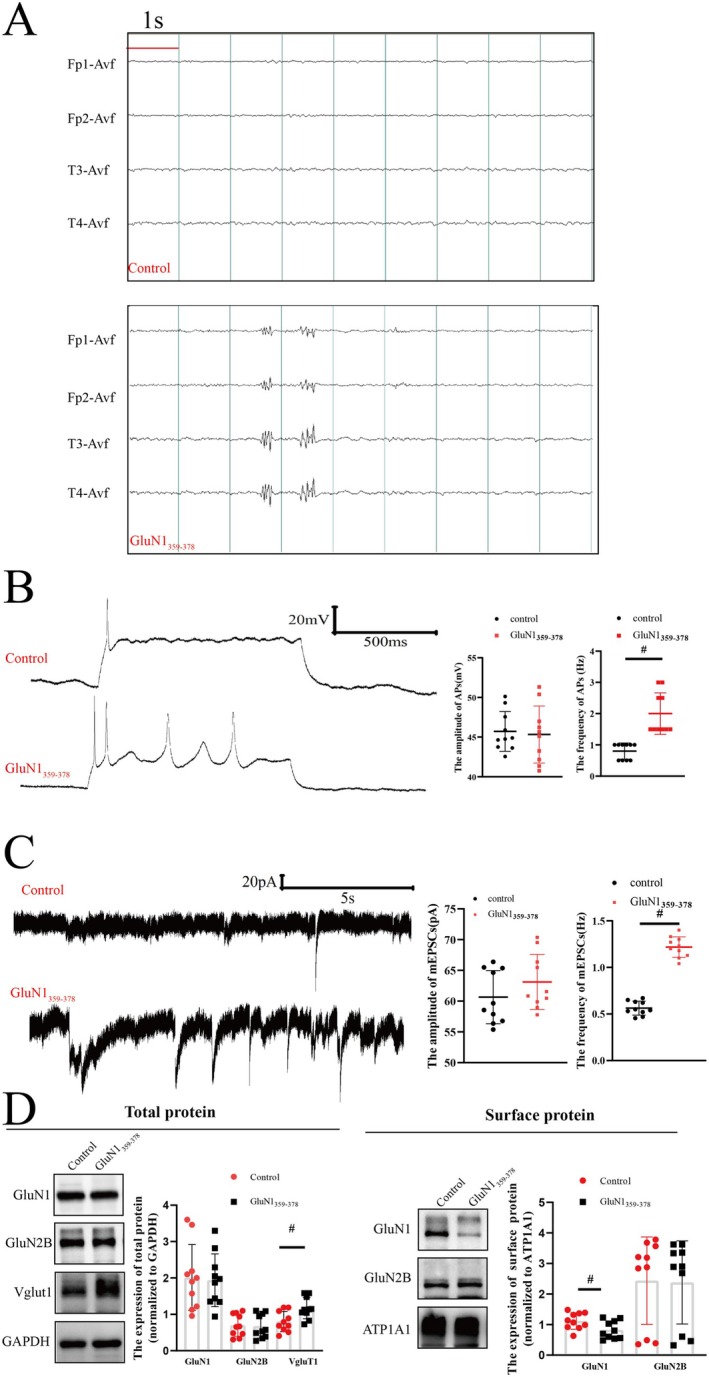
Neuro‐excitability was increased in mice with anti‐NMDAR encephalitis. (A) EEG results: No epileptiform discharges were recorded in mice in the control group, whereas epileptiform discharges were identified in mice in the GluN1_359–378_ group (time scale: 1 s). (B) Neuronal APs: The frequency of neuronal APs was significantly increased in the GluN1_359–378_ group (*n* = 10, half male and half female; *p < 0.05). (C) Neuronal mEPSCs: The frequency of neuronal mEPSCs was significantly increased in the GluN1_359–378_ group (*n* = 10, half male and half female; #*p* < 0.01). (D) Western blot: The expression level of total Vglut1 protein was increased in the GluN1_359–378_ group (*n* = 10, half male and half female; #*p* < 0.01). The expression level of surface GluN1 protein was significantly decreased (*n* = 10, half male and half female; *p < 0.05).

No significant symptoms of seizure were observed in mice in the GluN1_359–378_ group. Subsequently, the EEG of mice was recorded for 30 min every day, and the results showed that 80.00% (8/10) of the EEGs of mice in the GluN1_359–378_ group exhibited epileptiform discharges, whereas no epileptiform discharges were recorded in mice in the control group (Figure 2A). Thereafter, the APs and mEPSCs of neurons were assessed after incubation with serum. The results demonstrated that the frequency of neuronal APs was significantly higher in the GluN1_359–378_ group compared to the control group (*p* < 0.01, Figure 2B), suggesting increased neuronal excitability. Likewise, the frequency of mEPSCs was significantly higher in the GluN1_359–378_ group compared to the control group (*p* < 0.01, Figure 2C), indicating increased release of presynaptic glutamate. At the same time, the results of Western blot analysis uncovered that while the protein expression level of total GluN1 was comparable between the GluN1_359–378_ group and the control group, the expression level of surface protein GluN1 was significantly lower in the GluN1_359–378_ group (*p* < 0.05, Figure 2D). However, the expression of GluN2B protein and GluN2B surface protein remained unaltered (*p* > 0.05, Figure 2D). Additionally, the expression of Vglut1 (glutamate vesicular transporter 1), which reflects glutamate release, was determined. Western blot analysis indicated that the expression level of Vglut1 was higher in the GluN1_359–378_ group compared to the control group (*p* < 0.01, Figure 2D).

### 3.3 Increased Susceptibility to Seizures in Mice With Anti‐NMDA Receptor Encephalitis

**FIGURE 3:**
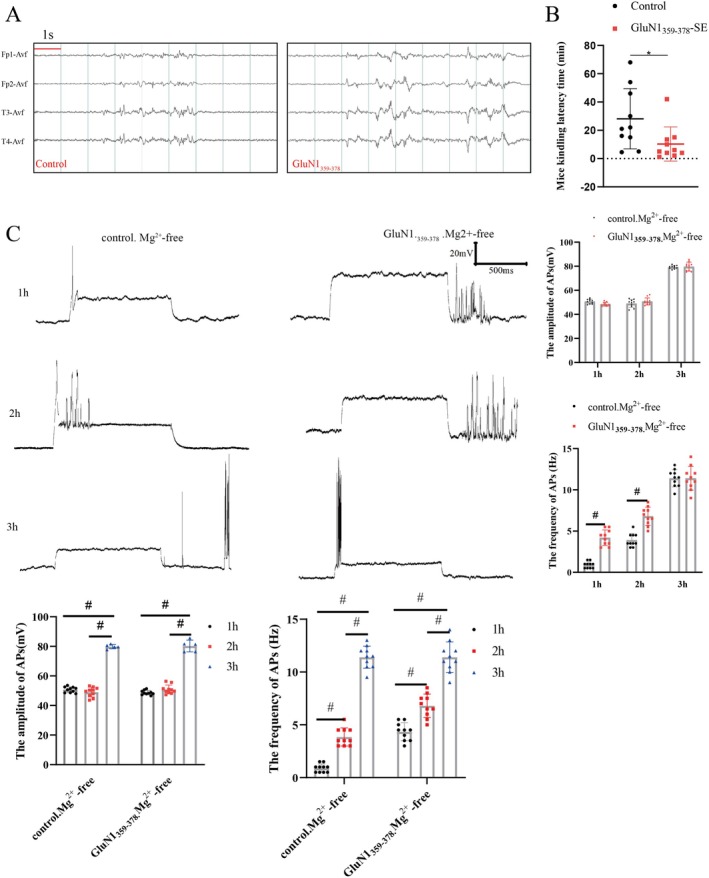
Increased susceptibility to seizures in mice with anti‐NMDAR encephalitis. (A) EEG of mice intraperitoneally injected with PTZ (time scale: 1 s). (B) Seizure latency was significantly shorter in mice in the GluN1_359–378_ group (*n* = 10, half male and half female; *p < 0.05). (C) Neuronal APs after exposure to Mg^2+^‐free solution: The frequency of neuronal APs in mice in the GluN1_359–378_ group was significantly increased after 1 and 2 h of exposure to Mg^2+^‐free solution (*n* = 10, half male and half female; #*p* < 0.01).

Mice in both the control group and the GluN1_359–378_ group had a 100% incidence of seizures after the administration of PTZ, accompanied by significant epileptic discharges in EEG recordings (Figure 3A). The latency period to seizure onset was 28.15 ± 21.30 min in the control group and 10.29 ± 12.08 min in the GluN1_359–378_ group (*p* < 0.05, Figure 3B). More importantly, at 1 and 2 h following exposure to the Mg^2+^‐free solution, the frequency of neuronal APs in the GluN1_359–378_ group was significantly higher compared to the control group (*p* < 0.01, Figure 3C). After 3 h of exposure to the Mg^2+^‐free solution, significant synchronous discharges were observed in both the control group and the GluN1_359–378_ group. Lastly, the frequency and amplitude of neuronal APs were similar between the control group and the GluN1_359–378_ group (*p* < 0.05, Figure 3C).

## 4 Discussion

As is well documented, anti‐NMDAR encephalitis is a CNS disorder caused by autoimmune dysfunction [1]. Given its unclear pathogenesis, there is a pressing need to construct a stable animal model. Previous studies have reported that rodents receiving cerebrospinal fluid or IgG antibodies derived from patients with anti‐NMDAR encephalitis exhibited depression‐like behavior, anhedonia, and memory deficits [22, 23]. However, these models are regarded as passive immune models that can only demonstrate the pathogenicity of NMDA receptor antibodies but fail to simulate autoantibody production [24]. Wagnon et al. demonstrated that an animal model constructed using the immunogenic peptide GluN1_359–378_ could simulate autoantibody production and is thus considered the closest approximation to real anti‐NMDAR encephalitis models [29]. Consequently, an animal model of anti‐NMDAR encephalitis was constructed based on Wagnon *I*'s method.

The success of model construction was initially evaluated based on clinical diagnostic criteria for anti‐NMDAR encephalitis. According to the criteria proposed by Grau, confirmed anti‐NMDAR encephalitis needs to fulfill the following three conditions [32]: (1) presence of one or more of the six chief symptoms: (i) abnormal (psychiatric) behavior or cognitive dysfunction; (ii) speech dysfunction; (iii) seizures; (iv) movement disorder, including dyskinesias, rigidity, or abnormal posture; (v) decreased level of consciousness; (vi) autonomic dysfunction or central hypoventilation. (2) Detection of positive NMDAR‐Ab: Cell‐based assays (CBA) are used to detect positive antibodies in CSF. If only serum samples are available for detection, positive CBA results, along with tissue‐based assays (TBA) and indirect immunofluorescence assays (IIF) of cultured neurons, are used for final diagnosis. (3) Exclusion of other diseases. Herein, despite not monitoring the animals for 24 h, EEG recordings depicted epileptiform discharges in mice in the GluN1_359–378_ group, suggestive of seizure activity. Following this, the presence of NMDAR antibody was evaluated. Given that mouse serum was utilized, CBA, TBA, and IIF experiments were performed on neurons to evaluate modeling efficacy. ELISA results indicated that the serum collected from mice in the control group was positive for NMDAR‐Ab, whereas serum acquired from those in the GluN 1_359–378_ group was negative for NMDAR‐Ab. Previous studies have documented the potential for false positives and false negatives in serum NMDAR‐Ab testing [33, 34]. In the present study, the positive expression in the hippocampal tissue of mice incubated with the serum collected from mice in the GluN1_359–378_ group was the most pronounced, indicating positive TBA results. Next, the evaluation of HEK937T cells transfected with NMDAR subunits showed positive CBA results with the serum collected from mice in the GluN1_359–378_ group. Finally, IHC and IIF were conducted on neurons, unveiling significantly positive results for neurons incubated with the serum from mice in the GluN1_359–378_ group. Based on EEG findings and multiple NMDAR‐Ab detection assays, a stable anti‐NMDAR encephalitis model was successfully constructed.

EEG recordings of mice receiving GluN1_359–378_ showed epileptiform discharges, suggesting increased neuronal excitability. Then, neurons exposed to mouse serum were examined via patch clamp electrophysiology. The frequency of neuronal APS in the GluN1_359–378_ group was increased, suggesting that GluN1_359–378_ increased neuronal excitability. Of note, earlier studies concluded that anti‐NMDAR encephalitis largely results in a decrease in NMDAR expression on neurons [19, 20]. To further investigate the mechanism underlying neuronal hyperexcitability, neuronal mEPSCs were further examined, revealing that the amplitude of neuronal mEPSCs was significantly increased, suggesting increased release of presynaptic Glu, whereas the amplitude of mEPSCs, representing postsynaptic excitatory receptor activity, was not significantly altered. It is worthwhile emphasizing that Western blot analysis indicated that the expression level of Vglut1 was increased, suggesting the enhanced release of presynaptic Glu, which was consistent with the results of patch‐clamp electrophysiology. Despite the decreased expression level of GluN1 on the neuronal surface, the expression level of GluN2B was not significantly changed, indicating a shift in the composition of NMDARs on the neuronal surface. The amplitudes of mEPSCs largely reflect the activity of glutamate receptors on the postsynaptic membrane, which principally comprises α‐amino‐3‐hydroxy‐5‐methyl‐4‐isooxazole receptors (AMPARs) [21], mediating over 90% of rapid excitatory synaptic transmission in glutaminergic synapses [35]. Although the immunogenic peptide GluN1_359–378_ altered the composition of NMDARs on neuronal surfaces and compromised their function, AMPARs predominantly mediated neural excitatory function, which might have accounted for the unaltered mEPSC amplitude. Finally, in order to determine the effect of the immunogenic peptide GluN1_359–378_ on seizures, in vivo and in vitro seizure models were constructed. Following an intraperitoneal injection of PTZ, the seizure latency of mice in the GluN1_359–378_ group was significantly reduced, suggesting that GluN1_359–378_ increased their susceptibility to seizures. Noteworthily, neurons exposed to the Mg^2+^‐free solution for 3 h exhibited significant synchronous discharges, which are widely recognized as an in vitro model of seizure [36]. However, no significant synchronous discharges were detected in neurons exposed to the Mg^2+^‐free solution for less than 3 h [37]. After incubation with serum derived from mice in the GluN1_359–378_ group, an increase in the frequency of neuronal APs was observed at both 1 and 2 h after exposure to Mg^2+^‐free solution. However, there was no significant difference in the amplitude and frequency of neuronal APs after 3 h of exposure to the Mg^2+^‐free solution between the control group and the GluN1_359–378_ group. Taken together, these results collectively suggested that the immunogenic peptide GluN1_359–378_ increased susceptibility to seizures without impacting their severity. No overt sex‐specific differences were detected in seizure susceptibility, neuronal excitability, or mEPSC frequency. Nevertheless, some limitations in this study warrant acknowledgment. To begin, although sex distribution was balanced in the experimental design, the small subgroup sizes precluded robust statistical comparisons. Secondly, patch clamp analysis of brain slices was not conducted on the mouse models. Thirdly, due to operational limitations, CSF was not extracted from mice to test for NMDAR‐Ab. Finally, a comprehensive evaluation of the behavior of the mice was not performed.

## 5 Conclusion

To the best of our knowledge, this is the first study to demonstrate that increased glutamate release may be a potential mechanism underlying the susceptibility of patients with anti‐NMDAR encephalitis to seizures.

## Author Contributions

H.H. and Y.H. were responsible for the study, data collection, and data analysis. H.H., Y.H., and S.L. drafted the initial manuscript. Y.W. and S.Y. assisted in manuscript revision. Y.W. acquired funding and supervised the study. The final manuscript was read and approved by all authors. All authors contributed to the article and approved the submitted version.

## Ethics Statement

We confirm that we have read the Journal's position on issues involved in ethical publication and affirm that this report is consistent with those guidelines. All experimental procedures were approved by the Ethics Committee of Guangxi Medical University (ethical batch number: 202105005) and were conducted in accordance with the National Institutes of Health Guide for the Care and Use of Animals.

## Conflicts of Interest

The authors declare no conflicts of interest.

## Supporting information


**Data S1:** cns70585‐sup‐0001‐Supinfo.pdf.

## Data Availability

The data that support the findings of this study are available from the corresponding author upon reasonable request.
