# Supplementary figures and images for: Elevated Release of Presynaptic Glutamate: The Potential Pathogenesis of Anti‐NMDAR Encephalitis‐Associated Seizures
Source: CNS Neurosci Ther. 2025 Oct 17;31(10):e70585. doi: 10.1111/cns.70585 (PMC12531711; doi:10.1111/cns.70585)

Full unedited gel for Figure 1

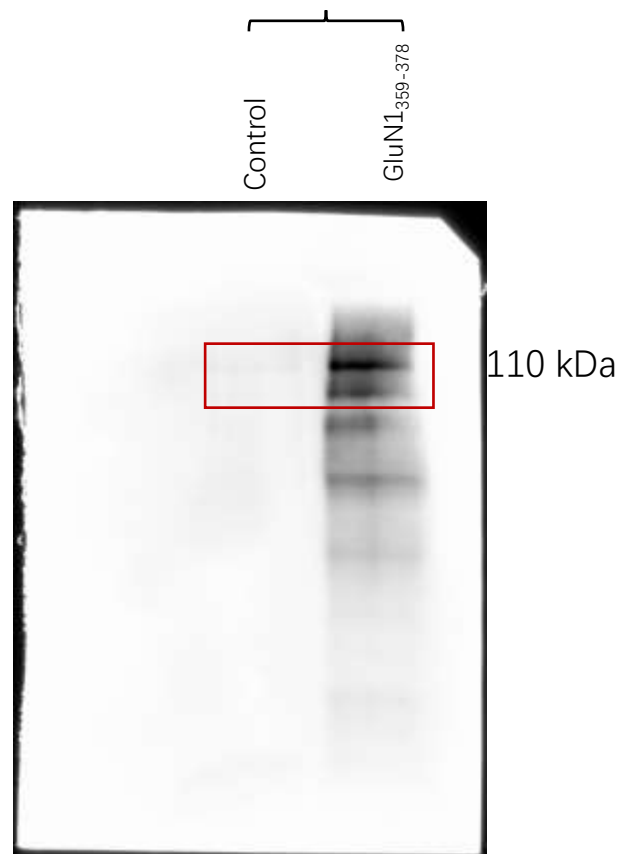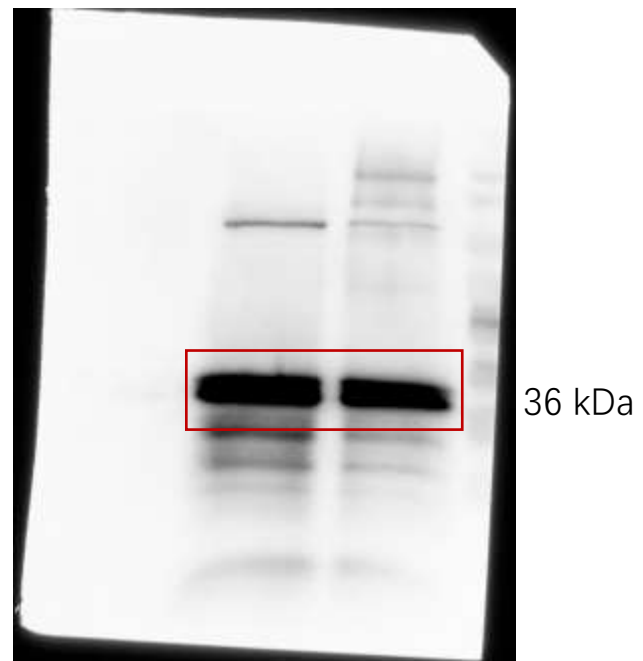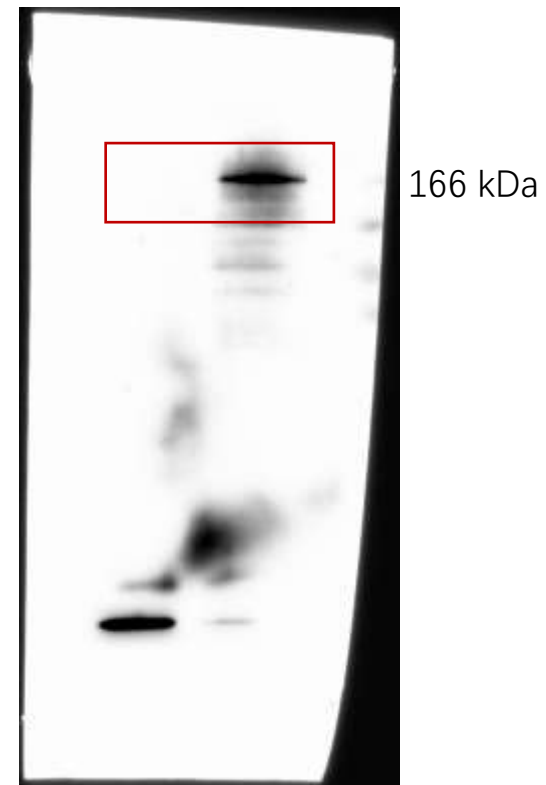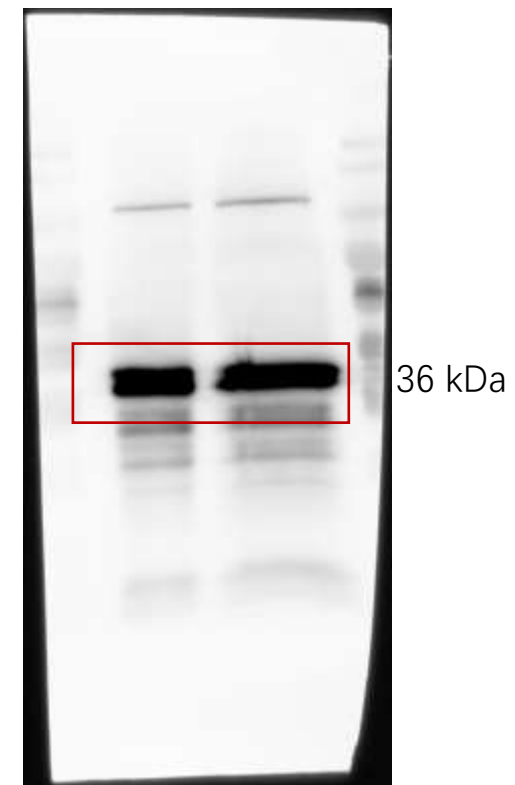

Full unedited gel for Figure 2

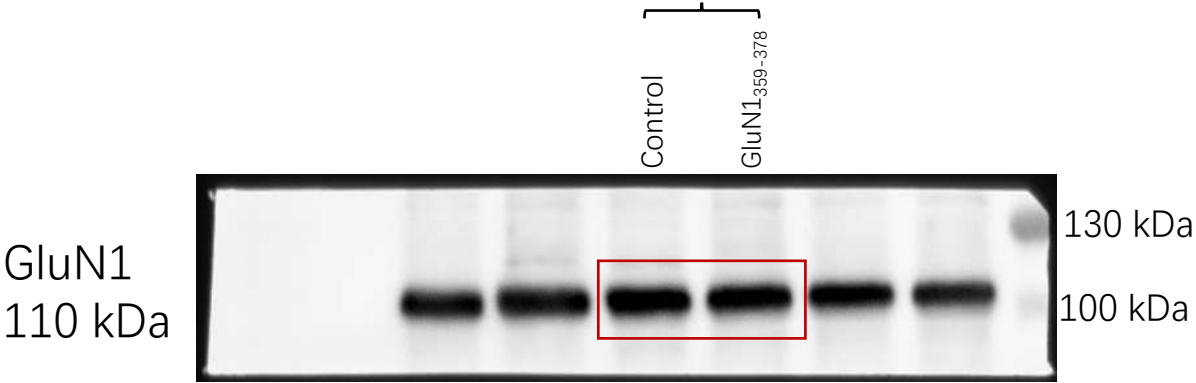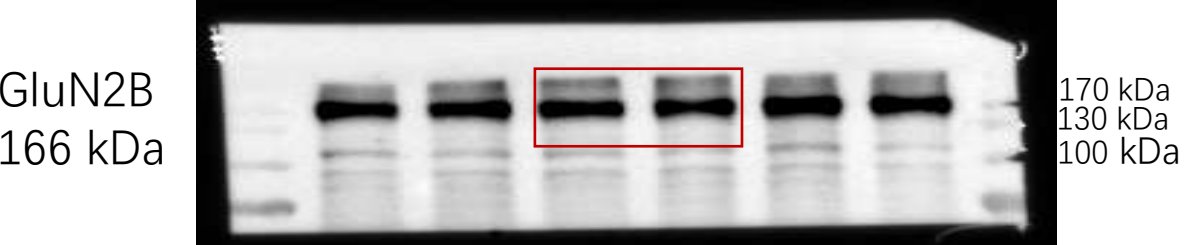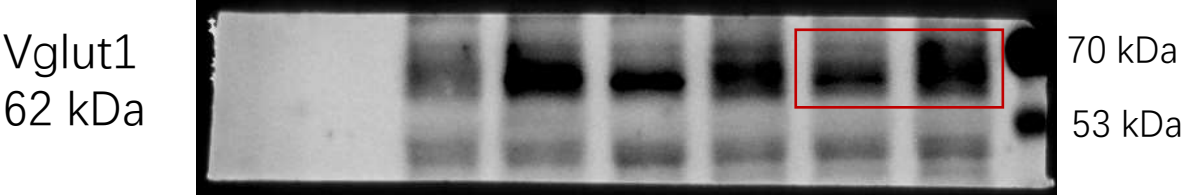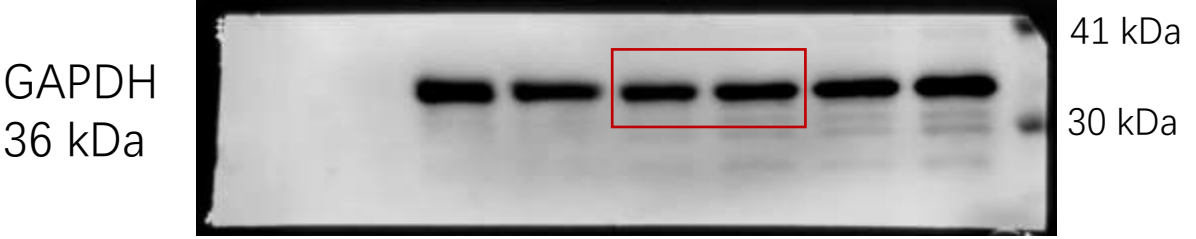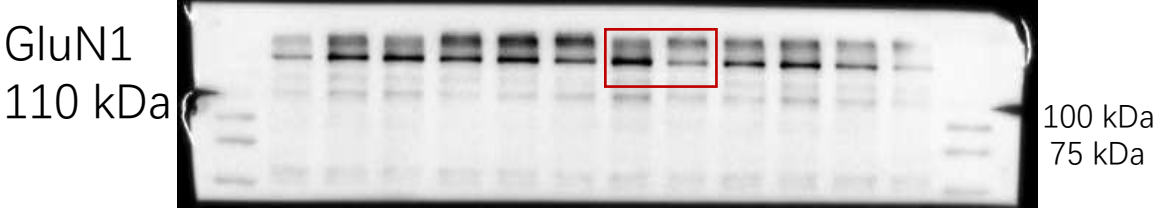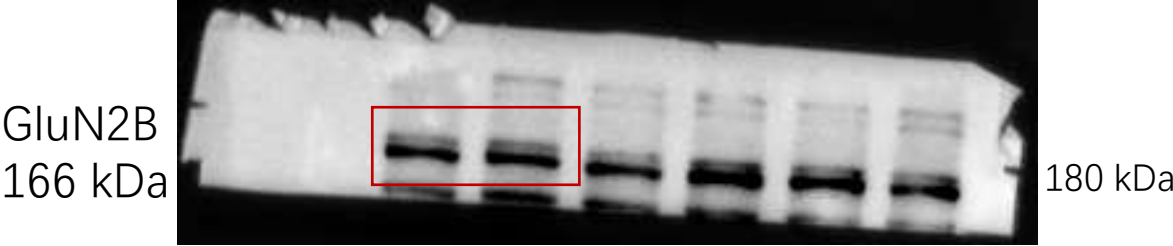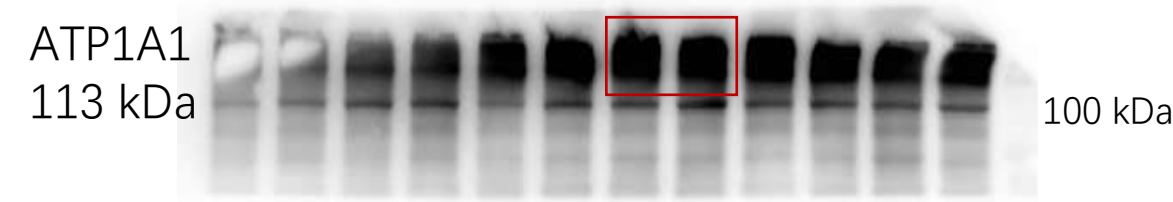

Supplement: Supplementary file 1 — Data S1: cns70585‐sup‐0001‐Supinfo.pdf. [file CNS-31-e70585-s001.pdf]
